# Feasibility and acceptability of conducting a birth cohort study during the COVID-19 pandemic: a mixed-methods study

**DOI:** 10.1186/s12884-025-07850-3

**Published:** 2025-07-16

**Authors:** Allison M. Grech, Nathalie Kizirian, Marjan Mosalman Haghighi, Sweekriti Sharma, Reeja Nasir, Roslyn Muirhead, Angelika Zankl, Clare Collins, Andrew Holmes, Adrienne Gordon

**Affiliations:** 1 Faculty of Medicine and Health, Central Clinical School, University of Sydney, Camperdown, NSW 2006 Australia; 2 Charles Perkins Centre, University of Sydney, Camperdown, NSW 2006 Australia; 3 Sydney Institute for Women, Children and their Families, Sydney Local Health District, Camperdown, NSW 2050 Australia; 4 Faculty of Medicine and Health, The University of Sydney, School of Public Health, Sydney Health Literacy Lab, Sydney, Australia; 5 Institute for Musculoskeletal Health, The University of Sydney and Sydney Local Health District, Sydney, Australia; 6 School of Health Sciences, College of Health, Medicine and Wellbeing, University of Newcastle, Callaghan, NSW 2308 Australia; 7 Food and Nutrition Research Program, Hunter Medical Research Institute, Rankin Park, NSW 2287 Australia; 8 Faculty of Science, School of Life and Environmental Sciences, University of Sydney, Camperdown, NSW 2006 Australia

**Keywords:** Pregnancy, Cohort studies, Feasibility, Acceptability, Pandemic, COVID-19, DOHaD

## Abstract

**Background:**

“BABY1000” was a pilot prospective birth cohort study based in Sydney, Australia, which aimed to identify factors before and during pregnancy that impact child and long-term health and assess study feasibility and acceptability. The COVID-19 pandemic and associated public health orders affected study protocols. This sub-study aimed to explore feasibility and acceptability of BABY1000 within this context.

**Methods:**

Recruitment of women commenced before conception or during early pregnancy attending clinics at a major hospital in Sydney. Data collection extended from recruitment, across pregnancy at 12-, 20-, 28- and 36-weeks’ gestation, and postnatally (in both mothers and children) until the child’s second birthday. Feasibility was assessed using routinely collected data on recruitment, retention, and completion. BABY1000 participants were invited to complete an online acceptability survey, including the State-Trait Anxiety Inventory, and to participate in focus group discussions. Paired t-tests were used to compare sub-study respondents to the wider cohort of participants to assess for demographic drivers of feasibility. Chi-squared tests were used to examine associations between high anxiety and demographic characteristics, study acceptability, and the perceived impact of COVID-19 on study participation.

**Results:**

From 225 women recruited, 180 (80%) remained enrolled at the end of pregnancy, with 100 (44%) remaining in the study until 24-months. Participant retention and data completeness were most challenging after birth. Eighty-seven mothers completed the acceptability survey and 22 parents participated in focus groups. Most participants found the study protocol to be acceptable and comfortable. A “high” anxiety score was not associated with study acceptability or willingness to participate, although metrics of both feasibility and acceptability were negatively impacted by the COVID-19 pandemic.

**Conclusions:**

Whilst public health orders associated with the COVID-19 pandemic impacted feasibility and acceptability of the BABY1000 cohort, this sub-study found it is both feasible and acceptable to collect comprehensive biological, questionnaire, and health data from early pregnancy to two years. Study designs which tailor resources to where participant attrition can be predicted and have capacity to adapt to changing circumstances will be best placed to contribute to understanding of the Developmental Origins of Health and Disease.

**Supplementary Information:**

The online version contains supplementary material available at 10.1186/s12884-025-07850-3.

## Introduction

The Developmental Origins of Health and Disease (‘DOHaD’) paradigm postulates that environmental factors in the first 1,000 days of life can impact disease risk in childhood, adulthood, and subsequent generations [1, 2]. Longitudinal birth cohort studies allow for comprehensive data collection over time and can help unravel the complex, multi-dimensional interplay of factors affecting human health and disease [3–5]. Taken at critical life stages, these rich data can be used to evaluate the impact of individual differences, family circumstances, social contexts, and national and international events on individuals, and, when extrapolated, populations. Whilst having greater investigative power, prospective birth cohorts are also prone to disruption from missing data. This is of particular relevance to cohorts addressing DOHaD hypotheses, which are already encumbered by the challenges of longitudinal research and can be logistically complex, expensive, and slow to generate and disseminate findings [6].

To understand associations between early exposures and subsequent health outcomes, birth cohort studies starting at preconception or early pregnancy are especially valuable. However, they are rarely performed, since studies involving women in the perinatal period are difficult to recruit to and conduct [7, 8]. Whilst recruitment before conception is possible, as shown by researchers within our own group [9] and other cohorts [10–13], almost 50% of pregnancies are unplanned [14] and not all women recruited when planning to conceive will achieve pregnancy. In pregnancy trials, it has been reported that approximately one third of eligible women typically consent to participate [8]. Retention can also be challenging; research involvement can be seen as inconvenient and/or interfering with the emotional, physical, and other adaptations associated with pregnancy and new parenthood [8, 15], impacted by geography [16, 17], and other factors. Whilst other prospective studies have described some of these reasons in their own cohorts in Norway, Denmark and the UK [18–20], the reasons for loss-to-follow-up can be unique across geographical populations and contexts. It is therefore imperative to continue to assess measures of feasibility and acceptability from birth cohorts to design future study protocols that are adapted to the needs of families and researchers alike.

Since early 2020, global COVID-19 pandemic management responses have exacerbated research challenges. Public heath orders designed to reduce disease transmission, such as periods of “lockdown”, alongside community concern regarding attending healthcare and/or other public settings, affected all aspects of human research, from participant recruitment and engagement to data collection and dissemination. Meanwhile, research institutions, teams and personnel were required to significantly reduce or cease onsite research and pivot to remote models, prioritise research relating to COVID-19, and/or abandon research plans to support frontline care or decision-making pertinent to the pandemic [21]. The impact of this context on the feasibility and acceptability of perinatal cohort research remains important to explore.

The pilot stage of the “BABY1000” longitudinal birth cohort study based in Sydney, Australia, collected comprehensive data at multiple timepoints throughout the first thousand days [22]. Although data collection was largely aligned with routine maternity care visits, the COVID-19 pandemic imposed substantial unforeseen challenges relating to recruitment, implementation, and follow-up, notwithstanding impacts on BABY1000 participants and investigators. One of the pre-defined measures within the study was an assessment of “state” anxiety, using the Spielberger State-Trait Anxiety Inventory 6-item short form (STAI-6) [23]. “State” anxiety refers to a temporary reaction in response to a perceived threat or stressful situation; in contrast to “trait” anxiety, associated with one’s general tendency towards anxiety [23]. It was hypothesized that those with higher STAI scores may be more likely to drop out of ongoing research or become lost to follow-up, given the context of the COVID-19 pandemic. Although previous systematic reviews have described an increase in state anxiety using the STAI tool in women in the perinatal period during pandemic [24–26], the influence of this on measures of research feasibility and acceptability have not, to our knowledge, been explored.

To that end, this manuscript: (1) assesses the feasibility and acceptability of BABY1000 design, using routinely collected data alongside feedback from participants, (2) explores the impact of COVID-19 and participant state anxiety on assessment of feasibility, acceptability and research participation, (3) describes the impact of the COVID-19 pandemic on feasibility and acceptability from the researcher’s perspective, and (4) provides recommendations for future research, based on learnings from the pilot BABY1000 experience.

## Methods

The BABY1000 pilot study was a prospective longitudinal birth cohort study based in Sydney, Australia, which enrolled those planning pregnancy or at 10–12 weeks’ gestation, with follow-up of both mother and child extending to the child’s second birthday. Participants were enrolled between December 2017 and August 2020; however, ‘active’ recruitment by researchers was conducted in 2018, 2019 and early 2020. Recruitment ceased earlier than planned due to research restrictions during the COVID-19 pandemic. Participants who were enrolled before 2018 and after March 2020 contacted study investigators independently and volunteered to participate. Supplementary Table 1 summarises maternal and infant samples, measurements, and questionnaires administered across the study timeline. Information regarding study design, protocol, measures, and statistical analyses has been previously published [22].

The acceptability analysis presented in this manuscript reflects the experiences of a subset of participants enrolled in BABY1000. All enrolled participants with ongoing pregnancies who had not withdrawn from the study (*n* = 144) were invited via email to complete an online questionnaire, followed by an invitation to participate in an online focus group discussion (FGD). Up to two reminder emails were sent to non-responders, two weeks apart. If no response was received after this time, no further contact was made regarding the sub-study.

### Study design

This sub-study used a mixed methods design, consisting of an online questionnaire (see Supplementary Material 1), FGDs (acceptability analysis), and review of routine data collected for the BABY1000 pilot study (feasibility analysis). Impacts of the COVID-19 pandemic on investigators were generated by discussion and consensus within the team.

#### Feasibility

Pre-defined measures of feasibility included:

- Number of participants recruited (as a percentage of total possible recruits).
- Incidence of pregnancy in those recruited at preconception.
- Incidence of pregnancy loss and/or termination.
- Number of live births with complete birth data available.
- Incidence of loss to follow-up.
- Percentage of participants who provided data at baseline and throughout the study.

#### Acceptability – online questionnaire

The online questionnaire was completed using Research Electronic Data Capture (REDCap) management software (hosted by the Sydney Local Health District). REDCap is a secure, web-based software platform designed to support data capture for research studies [27]. The online questionnaire was open for completion between September and November 2021, and was divided into three sections: (1) the experience and acceptability of the BABY1000 pilot study, (2) the effect of the COVID-19 pandemic on participants and their families, and (3) the Spielberger State-Trait Anxiety Inventory 6-item short form (STAI-6) [23]. This article focuses on study acceptability and includes results of the STAI-6 in relation to this; the impact of the COVID-19 pandemic on participants and families has been previously published [28].

Questionnaire prompts were designed specifically for use in this sub-study and included a range of closed and open questions. Closed questions used Likert scales (e.g., “How comfortable were you with providing the samples requested of you as a participant?” with a corresponding 5-point scale from “very uncomfortable” to “very comfortable”) and multiple-choice questions (e.g., “Can you tell us about how appropriate you felt about the length of the questionnaires?” with options “Too long/Too short/Just right”). Open questions were included to provide additional detail (e.g., “Do you have any further comments relating to how your participation in the BABY1000 pilot study was affected by the COVID-19 pandemic?”).

The questionnaire included the STAI-6 [23], a short-form of the original 20-point scale [29] used to measure state anxiety at the time of completion. This scale is a reliable indicator of anxiety during pregnancy [30]. Each item of the STAI-6 has four response categories (“not at all,” “somewhat,” “moderately,” and “very much”) corresponding to numerical values (1–4), with three of the six prompts negatively scored. Scores are summed to produce a total score ranging from 6 to 24. To derive sum scores comparable to the long-form tool, STAI-6 scores were divided by six and multiplied by 20, producing a possible range between 20 and 80 [31]. Sum scores above 40 were considered indicative of high anxiety [32].

#### Acceptability – virtual focus group discussions

Upon completion of the online questionnaire, participants were invited to contribute to a FGD. FGDs were hosted online via Zoom Video Communications, Inc. (“Zoom”) in November 2021. Participants were incentivised by a “Question and Answer” session on infant and toddler nutrition, hosted by a paediatric dietitian (AMG) following the session.

FGDs lasted 45–60 min and were audio-recorded and transcribed to allow detailed analysis of qualitative data. Discourse analysis [33] was used to explore the beliefs, values, and practices of participants. Analysis was completed independently by two researchers (AMG and SS) and any disagreements in coding were resolved by discussion. A list of themes and associated codes (or sub-themes) was generated, ensuring they captured the breadth of responses. Across the five FGDs, data saturation was reached for the themes identified.

### Statistical analysis

Descriptive analyses were conducted to present demographic data, identify proportions of responses to closed questionnaire prompts, and report the mean score and prevalence of state anxiety in our sample (using the STAI-6) according to pre-defined groups, as outlined above. Inferential statistics were conducted using IBM SPSS Statistics (version 28.0, Armonk, NY: IBM Corp). The level of significance was set at *P* ≤ 0.05. Paired t-tests were used to compare the subset (*n* = 88) to active cohort (*n* = 180) in Table 1. Chi-squared tests were used to examine associations between high converted STAI-6 scores (> 40) and parity, timing of birth (before or after February 2020, i.e., the start of the COVID-19 pandemic), participant assessment of study ease, and the impact of the pandemic on participation, including FGD involvement.

**Table 1:** Demographic characteristics of participants

Category^	Active cohort (*N*=180) N (%)	Participating sub-sample (*N*=88) N (%)	*P* value*
Age (years) at study entry			
Mean age (SD)	33.3 (4.2)	33.5 (3.8)	NS
20 – 29	23 (12.8)	12 (13.6)	
30 – 34	99 (55.0)	47 (53.4)	
35 – 39	45 (25.0)	22 (25.0)	
≥ 40	13 (7.2)	7 (8.0)	
Ethnicity			
European	103 (57.2)	57 (64.8)	NS
South Asian	18 (10.0)	5 (5.7)	
East Asian	23 (12.8)	9 (10.2)	
South East Asian	11 (6.1)	6 (6.8)	
Middle Eastern	4 (2.2)	2 (2.3)	
African	1 (0.6)	1 (1.1)	
Pacific Islander	4 (2.2)	1 (1.1)	
Other	12 (6.7)	6 (6.8)	
Missing ethnicity data	4 (2.2)	1 (1.1)	
Born in Australia			
Yes	96 (53.3)	53 (60.2)	NS
No	84 (46.7)	35 (39.8)	
Education – highest qualification			
Postgraduate Doctorate degree	12 (6.7)	7 (8.0)	NS
Postgraduate Master’s degree	34 (18.9)	19 (21.6)	
Undergraduate degree	72 (40.0)	33 (37.5)	
Non–university vocational training	20 (11.1)	10 (11.4)	
High school completion	5 (2.8)	3 (3.4)	
Less than high school completion	2 (1.1)	1 (1.1)	
Missing education data	35 (19.4)	15 (17.0)	
Previous live births			
0	104 (57.8)	51 (58.0)	NS
1	65 (36.1)	30 (34.1)	
2	8 (4.4)	6 (6.8)	
≥ 3	3 (1.7)	1 (1.1)	
Medical pregnancy complications			
None recorded	133 (73.9)	68 (77.3)	NS
Gestational diabetes	31 (17.2)	15 (17.0)	
Hypertensive disorders of pregnancy	5 (2.8)	2 (2.2)	
Other	11 (6.1)	3 (3.4)	
Timing of birth			
June 2018 – end of January 2020	101 (56.1)	46 (52)	NS
February 2020 – July 2021	79 (43.9)	42 (48)	

^“Active cohort” reflects those who were continuing in the study at the birth of their child; “Sub-sample” refers to those participants who completed the online survey

*Paired t-tests assuming unequal variances used to calculate *P*-values for age (continuous variable). Chi – squared test used to determine *P*-values between groups for remaining categories (all categorical variables – European vs. other ethnicities; born in Australia vs. elsewhere; University-level education vs. below; Primiparous vs. multiparous; No medical complications in pregnancy vs. any complication; Timing of birth of child – pre-COVID-19 vs. during COVID-19). All associations were non-significant (NS)

### Ethics

BABY1000 had ethical approval from Sydney Local Health District (RPAH zone) Review Committee. This sub-study received additional ethics approval to include questions relating to the impact of the COVID-19 pandemic. All participants who agreed to complete the questionnaire were provided information about sub-study procedures, benefits, voluntary participation, confidentiality, and complaints processes, and were required to indicate they had read this prior to consenting to involvement.

## Results

### Sample numbers and characteristics

**Fig. 1:**
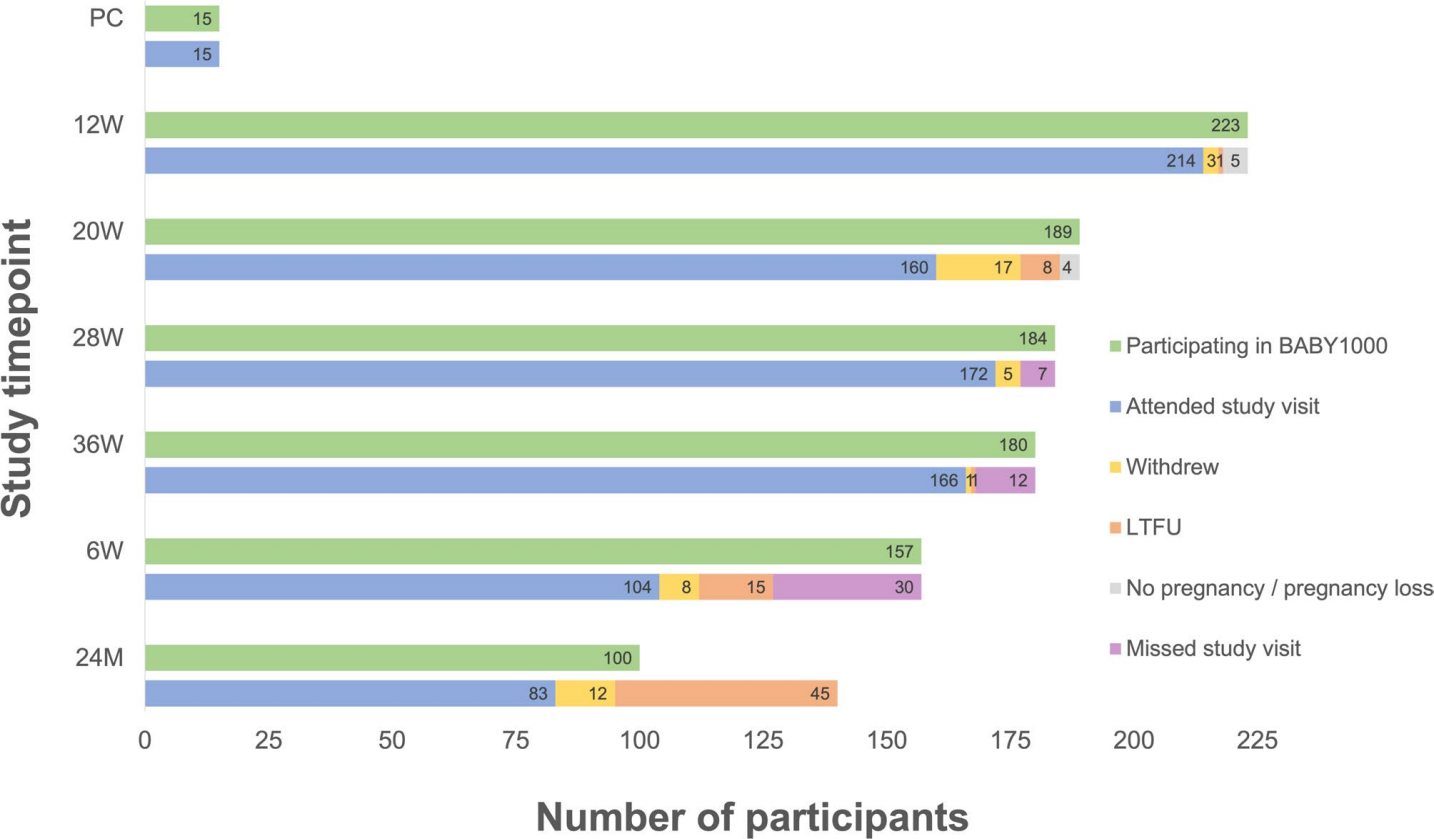
BABY1000 participant retention, study visit attendance and reasons for non-attendance. PC = Preconception; W = weeks; M = months; LTFU = lost to follow-up. The total number of study participants at 24 M (*N* = 100) was calculated by subtracting the sum of those who withdrew (*N* = 46), were LTFU (*N* = 70), or did not achieve pregnancy or had a pregnancy loss (*N* = 9) from the total number recruited (*N* = 225)

A total of 225 participants were enrolled in the BABY1000 pilot study. Most women were recruited in the first trimester (*n* = 210), with a subset recruited preconception (*n* = 15, including one participant who enrolled at preconception in two pregnancies). At the end of pregnancy, 180 participants remained enrolled (80%). Further detail outlining study completion has been previously published [22]. A final 83 participants (including 84 children) completed the study to 24 months, from 100 still enrolled at the end of the study period (Fig. 1).

Demographic characteristics of participants are summarised in Table 1. Participants were broadly representative of birthing women regionally in terms of age, parity, country of birth, ethnicity, and BMI [22]. The mean age of mothers at recruitment was 33.5 years. Most were primiparous (57%), born in Australia (60%) and identified as being of European background (65%). Unfortunately, we were not able to recruit women identifying as Aboriginal or Torres Strait Islander peoples, who comprise 3.8% of the Australian population [34]. Approximately half (52%) of babies were born prior to the beginning of the pandemic. Whilst they were more likely to have been born in Australia and identify as being of European ethnicity, participants who elected to participate in the sub-study were not significantly different to the “active” cohort in relation to the demographic characteristics measured.

### Feasibility

#### Number of participants recruited (as a percentage of total possible recruits)

A total of 225 participants were enrolled between December 2017 and August 2020. Active recruitment was conducted from February 2018 to March 2020; those enrolled before 2018 and after March 2020 contacted the study team and volunteered to participate. Recruitment was ceased in March 2020 due to the COVID-19 pandemic. In total, 60 participants (21% total recruited) were recruited in 2018, 152 (68%) in 2019, and 10 (4%) in 2020. An additional three participants volunteered after recruitment had ceased and two mothers participated twice.

The number of 12-week ultrasound appointments made in the clinic for each year were as follows: 281 in 2018, 776 in 2019, and 223 in January-March 2020, representing 1280 potential recruits. However, research staff worked part–time, actively recruiting up to three days a week. We estimated that a maximum of 768 women were eligible for the study over the time period (assuming three days a week of potential recruitment) and therefore the estimated recruitment rate was 225 recruited women/768 potential recruits = 29%. Notably, not all scan attendees were eligible (e.g., not English-speaking, very high-risk pregnancy, etc.) and scan appointment numbers did not account for cancellations or sickness of staff members and/or recruiters, such that the “real world” recruitment potential would have been less. Unfortunately, we do not have access to further detail regarding this to estimate a denominator with greater precision.

Recruitment to BABY1000 was also impeded by several other obstacles. Clinic sonography staff and equipment were unavailable for four months in 2018, affecting recruitment logistics. BABY1000 staff also worked across multiple research projects, and at times, active recruitment was paused to focus on other projects. Recruitment ceased earlier than planned owing to impacts of the pandemic, including mandated pauses on research not related to COVID-19 and researchers predominately working from home as per Health and University directives.

#### Incidence of pregnancy in those recruited at preconception

From 15 participants (14 different women) recruited at preconception, 8 (53%) became pregnant during the study period.

#### Incidence of pregnancy loss and/or termination

Following enrolment, 1.4% of participants had a pregnancy loss or termination.

#### Number of live births

A total of 209 live births across 206 pregnancies (including three sets of twins) were documented from 225 participants. Of the 19 participants who did not have live births recorded, seven did not fall pregnant, four experienced pregnancy loss, and eight did not have a verified child date of birth (lost to follow up). Complete birth data was available for 208 babies.

#### Incidence of loss to follow-up

By the end of the study at 24 months, a total of 70/225 participants (31%) were lost to follow up. Sixty of these (86%) occurred after the final study visit during pregnancy.

#### Percentage of participants who provided data at baseline and throughout the study

Table 2 shows study visit attendance and completeness of bio-sample data collection. At the last pregnancy visit (36-weeks), 166/180 (93%) of participants still enrolled in the BABY1000 study attended the study visit, representing 80% of total recruited participants. This declined to 83 (37% total recruited) by the last study visit around the child’s second birthday. A total of 100/225 (44%) participants provided data at study entry (preconception or 10–12 weeks), throughout pregnancy and at 24-months. Twenty-seven (12%) participants completed every timepoint study visit and BABY1000 questionnaire (not including the Australian Eating Survey (AES)), whilst only one participant attended each study timepoint visit and completed every questionnaire (including the AES). There was no association between timing of birth (before or during the COVID-19 pandemic) and attendance at the last study visit (*P* = 0.50).

**Table 2:**
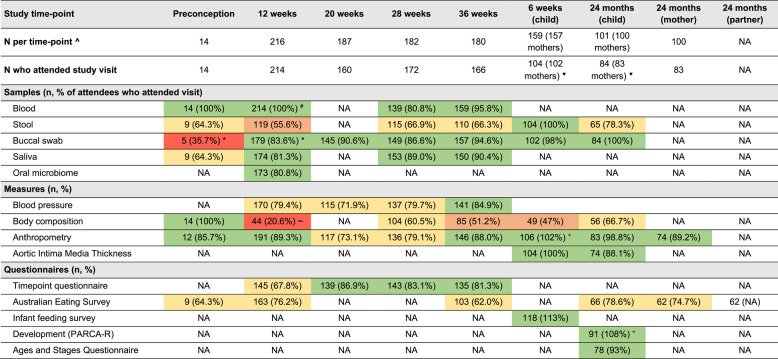
BABY1000 pilot study completion data for samples, measures, and questionnaires. Cells shaded in green reflects excellent completion (>80%), yellow reflects good completion (60-80%), amber reflects average completion (40-60%) and red reflects poor completion (<40%)

*NA* Not applicable

^^^Total number of participants who were still “in” the BABY1000 study at the corresponding time-point (i.e., excluding participants who did not become pregnant, withdrew, or were lost to follow up by that time-point)

^♥^Two sets of twins were included in the pilot study

^*^Buccal swab kits had not yet arrived when the study began recruiting, explaining poor completeness at preconception/12-week timepoints relative to other timepoints

^#^Blood was routinely collected for all participants as part of clinical care at the 12-week antenatal visit, however, for some participants, an additional blood draw was required for study purposes as this had not been combined with the routine blood collection – in this group only 87 (40.7%) consented; ~ Poor completeness of body composition (BOD POD) measurement at the 12-week timepoint is likely a combination of three factors: recruitment site (some participants were recruited at a hospital site, rather than in the clinic where the BOD POD machine was located), long appointment times, and a lack of prior warning for participants, such that they were not always wearing clothing suitable for a body composition scan

^+^Note that some participants completed questionnaires online or via post, but did not necessarily attend the corresponding time-point study visit

Notably, due to the COVID-19 pandemic and associated restrictions on face-to-face study visits, some planned visits and associated data were omitted from the pilot protocol. This included the removal of a study visit at 12 months, and for some participants, the use of routinely collected health data (e.g., anthropometry) in lieu of data collected by researchers, and omission of additional study measures usually collected. Other study data was attempted to be retrieved via post, however these were often lost or not returned. Complete data collection was therefore not possible for some participants, even if they were prepared to participate fully.

### Participant-rated acceptability

Of 180 active BABY1000 pilot study participants invited, 88 (49%) completed the online questionnaire. From these, 35 (40%) consented to participate in FGDs. Due to scheduling limitations, 22 parents (21 mothers and 1 father) participated in one of five virtual FGDs.

Participant acceptability of data collection is shown in Table 3. Most (> 80%) participants reported feeling “very comfortable” or “comfortable” with the collection of all study samples and measures, except for stool (75% for infant and 62% for maternal stool sampling). In FGDs, some participants reported feeling *“embarrassed”* or *“nauseated”* by taking stool samples (Supplementary Table 1). Additional feedback from FGDs relating to sample collection (e.g. stool, saliva) reflected the ease of sample collection (especially at-home collection), whilst others suggested that further personalised instruction and/or feedback was needed.

**Table 3:** Participant acceptability of maternal and child sample and measures collection, as measured by online questionnaire (total *n* = 87)

	Acceptability (comfort) assessment			
	Very comfortable / comfortable N (%)	Neutral N (%)	Uncomfortable / very uncomfortable N (%)	N^
Samples				
Blood (mother)	70 (84)	9 (11)	4 (5)	83
Stool (mother)	51 (62)	14 (17)	17 (21)	82
Stool (infant/child)	60 (75)	13 (16)	7 (9)	80
Saliva (mother)	76 (87)	6 (7)	5 (6)	87
Buccal swab (mother)	76 (87)	6 (7)	5 (6)	87
Measures				
Anthropometry (mother)	80 (92)	3 (3)	4 (5)	87
Anthropometry (infant/child)	79 (93)	2 (2)	4 (5)	85
Body composition – Bod Pod (mother)	70 (86)	7 (9)	4 (5)	81
Body composition – Pea Pod (infant)	50 (85)	4 (7)	5 (8)	59
Body composition – Bod Pod (child)	28 (82)	2 (6)	4 (12)	34
Developmental assessment (child)	34 (85)	2 (5)	4 (10)	40
aIMT* (child)	25 (83)	2 (7)	3 (10)	30

^N is variable since not all participants had completed the study when they completed the questionnaire or may not have consented to providing that sample or measure

*aIMT aortic intima media thickness, as measured by ultrasound and used as a marker of preclinical atherosclerosis

Acceptability of questionnaire administration in BABY1000 was mixed. The vast majority (91%) of participants reported feeling “very comfortable” or “comfortable” with questionnaire completion. However, common qualitative feedback was that some questionnaires were too long or difficult to complete, especially if administered postnatally (in the context of parenting responsibilities, often during COVID-19 lockdowns) and/or were unfortunately sent asynchronous to their child’s development (Supplementary Material 3). Others reported that online questionnaire completion was preferred and may have improved completion rates, particularly for longer questionnaires. Four participants commented that completing the questionnaires was interesting and provided an opportunity to *“learn about my baby a little bit more”* and *“consciously”* reflect on their development. The AES was sent via an online link and 92% of respondents reported feeling “very comfortable” or “comfortable” with its completion. However, free-text feedback relating to the AES critiqued its length, clarity, and ability to accurately reflect dietary intake, especially for those following a vegetarian diet, e.g., *“… we’re vegetarians*,* and we found that… some questions were kind of irrelevant.”*

Participants also reflected on the benefits of BABY1000 participation. As indicated by ten participants in FGDs (45% of those who participated), a major reported benefit of study involvement was access to an additional pregnancy scan at 28-weeks and the provision of individual medical feedback at study visits in relation to their babies, e.g. *“… having an extra person to give you feedback on how you’re progressing with your baby*,* I like that a lot”*. Others were motivated by the hope that their participation could help others: *“I liked idea of contributing to something…that my contribution might change something in the future.”*

Finally, participants reported that the COVID-19 pandemic and associated regulations caused BABY1000 protocol changes that were undesirable. One parent reported that she “*missed out on a few things”* that she *“… would have been very interested to participate in”* and another felt *“disappointed”* that an additional pregnancy scan was not possible due to restrictions on face-to-face contact with researchers. Others described that even when visits could resume, the risk of COVID-19 made participation less comfortable (e.g. *“… Hospital & centre felt a bit eerie with no-one around and all the extra check points”*) or was a barrier to attendance (e.g. *“Despite wanting to participate in the 2-year development assessment in person*,* I was not comfortable to attend the hospital due to COVID risk.”*) Although information provided by researchers was described as respectful and clear, more instruction and feedback regarding data collection was also requested, e.g. *“I felt like there was a lot of measurements taken but I wasn’t really sure what they meant or whether that was a healthy score…”* alongside more detail on how the study was affected by the pandemic *(“…it might be interesting to receive updates on what’s happening… at what stage*,* it is like is it progressing is it on pause or anything…”)*.

### Participant state anxiety (STAI-6)

Of the 87 participants who completed the STAI-6, 39 (45%) had converted scores above 40, indicative of high anxiety. The mean score was 40 (SD 12.3), with scores ranging from 20 to 70. High STAI-6 scores (> 40) were significantly associated with having at least one medical pregnancy complication (χ^2^ = 7.0; df = 1; *P* = 0.008) but were not associated with indicators of acceptability, including overall study ease of participation (χ^2^ = 0.16; df = 1; *P* = 0.69), impact of COVID-19 on willingness to participate (χ^2^ = 0.04; df = 1; *P* = 0.85), or consenting to FGD participation (χ^2^ = 0.04; df = 1; *P* = 0.84).

### Feasibility and acceptability in the context of COVID-19– the researcher experience

The COVID-19 pandemic forced the BABY1000 team to navigate unforeseen challenges. Several staff members were clinician researchers required to focus on responding to the pandemic in their clinical areas. Researchers prioritised preserving study protocols whilst adhering to pandemic regulations on a limited budget. Consequently, protocol changes were required, notably cessation of in-person data collection in favour of sample collection via mail or online. This resulted in increased time spent following up participants to return self-collected study samples/measurements that were previously efficient to collect in-person. Cancellation of study visits and reduced contact with colleagues also affected staff morale.

When face-to-face visits could resume, precautionary measures including respiratory screening, ensuring participants were tested for COVID-19 prior to entry (as per NSW Health regulations), and communicating study protocol changes with participants resulted in more time spent arranging and conducting study visits. Researchers also found building rapport with children whilst wearing a mask difficult. These additional demands on researchers, alongside funding constraints and increased researcher absenteeism (due to infection or respiratory symptoms, public health orders, etc.) further reduced capacity for research to proceed normally.

### Learnings from the BABY1000 experience

Whilst the acceptability and feasibility of the pilot BABY1000 protocol was impacted by unforeseen effects of the COVID-19 pandemic, areas for improvement in three major areas were identified: recruitment, retention and data completeness, and study cost-efficiency. Recommendations and proposed benefits for each of these areas are summarised in Table 4 and explored in further detail in the Discussion.

**Table 4:** Recommendations for improving feasibility of future birth cohort research design, based on the pilot BABY1000 study experience

Issue or observation	Recommendation/s for future research	Proposed benefits
Recruitment		
• Recruitment was slow	• Schedule “waves” of recruitment • Complete data entry and checking between recruitment waves • Collect data on reasons for non-participation of eligible women as recruitment is conducted • Establish and maintain strong relationships with healthcare staff and management (if aligning with healthcare pathways)	• Focused attention on recruitment • Prevents researcher burn-out associated with recruitment when focusing on other tasks concurrently • Ensures consistency and accuracy of data reporting • Allows for early identification and amendment of issues with study protocol, data entry processes, and/or recruitment strategies • Greater support and cooperation from healthcare staff in identifying (and retaining) potential participants
Retention and data completeness		
• Data completeness was best when aligned with healthcare visits	• Embed research into existing healthcare pathways	• Improved data completeness – both through collection at the time of study visit and through data linkage with medical records
• Participant retention post-pregnancy was challenging	• Provide incentives for study visits, especially if scheduled outside of routine healthcare • Make study visits as easy as possible for participants, e.g. parking permits, flexible visit times, videoconferencing if required, etc. • Foster greater connection to the study, researchers and other participants, e.g. face-to-face events, opportunities to collaborate with researchers on research priorities or sub-study design	• Improved data completeness • Improved engagement and acceptability from a participant’s perspective – more likely to attend study visits outside of routine health appointments
• Face-to-face study visits were not always possible, even though they were preferred by both participants and researchers	• Offer videoconferencing as an alternative or adjunct to face-to-face visits • Use existing health data if available via electronic medical records • Use mail to collect samples, with clear written instructions and the option for clarification via telephone or videoconference if required	• Improved data completeness • Improved acceptability from a participant’s perspective
• Participants reported a lack of timely feedback regarding research findings, affecting engagement with the study	• Provide periodic written updates of research progress and outputs, e.g. via newsletters	• Improved participant understanding, engagement and retention • Improved researcher communication skills
• Time spent by research team following up data from participants was high	• Use apps to centralise data collection and systems, e.g. links to study questionnaires, embed automated prompts to complete data collection at defined time-points, provide alerts when new research is published	• Reduced researcher burden • Stronger connections between research team and participants • Allow for analytics – process evaluation • Improved acceptability and data completeness
• Questionnaires criticized as too long, hard to remember or misaligned with desired timing	• Offer questionnaires online, ensure they are optimised for mobile use • Send shorter, more frequent questionnaires across the study duration • Use automated systems for questionnaire administration and reminding • Offer to facilitate questionnaire completion via telephone interview with a researcher if helpful	• Improved data completeness and accuracy • Improved acceptability from a participant’s perspective
Study efficiency		
• Researcher time spent storing and analysing some biosamples was high	• Outsource bio-sample data analysis where feasible, e.g. gut microbiome data	• Reduced researcher burden • Improved efficiency of results generation • Strengthen links between research team and industry
• Budget constraints affected capacity to pivot in the context of adversity (i.e. COVID-19 pandemic)	• Consider the limits of research objectives in the context of available funding/resources • Consider collecting fewer samples or measures, focusing on quality and completeness • Consider designing student projects to answer discrete research objectives	• Funding availability is aligned with research goals • Supports student learning whilst generating study outputs

## Discussion

The BABY1000 pilot study provides evidence of feasibility and participant-rated acceptability of sample collection, measurements, and questionnaire administration across the first thousand days. However, despite having a lower prevalence of COVID-19 in Australia than many other high-income countries, public health orders considerably affected the research experience of many participants in our study, alongside researchers. Results regarding study feasibility and acceptability should be interpreted within this context.

Since the COVID-19 pandemic was likely to impact participant anxiety, which could affect feasibility and acceptability, the inclusion of the STAI-6 in our sub-study is a strength. A 2020 systematic review and meta-analysis showed that in pregnant and postpartum women, pooled total STAI scores, and the percentage of women with scores > 40, were significantly higher during pandemic [25]. Importantly, whilst the pandemic affected study feasibility (from both public health directives and participant concern), we found no association between high anxiety and study acceptability, willingness to participate, or consenting to FGD participation.

### Feasibility of BABY1000 and recommendations for future research

#### Recruitment

Recruitment was challenging for BABY1000. Although a well-known difficulty for birth cohort studies [7] and clinical trials recruiting perinatal women [8], difficulty recruiting can lead to responder bias, insufficient sample sizes, increased costs, delayed dissemination of findings, impacted researcher morale and premature cessation of recruitment or research projects entirely [35], highlighting the need to identify ways to improve recruitment methods.

Recruitment challenges differ for those planning pregnancy compared to those who are already pregnant. For those in the “preconception” stage, recruitment via dedicated pregnancy planning clinics is possible (as with BABY1000), but biased; worldwide, almost 50% of pregnancies are unplanned [14] and unplanned pregnancies are associated with a higher rate of adverse maternal and infant outcomes [36]. Further, not all women recruited at preconception will achieve pregnancy; in BABY1000, half of those recruited at preconception did not fall pregnant during the study period. Recruitment for pregnancy trials is consistently reported as slower than anticipated, with approximately one third of eligible women typically consenting to participate [8]; similar to our experience. For those who did not consent, data was not always collected regarding reasons why. This, plus ethical restrictions concerning contacting non-consented women, precluded an in-depth analysis of non-participation.

Recruitment to BABY1000 was further affected by challenges concerning clinic access, researcher availability and restrictions related to the COVID-19 pandemic. The pilot may have benefited from scheduled “waves” of recruitment that could allow for data checking and entry between waves, maintain researcher motivation, and ensure forward progress with other research tasks. However, in a pandemic situation affecting face-to-face recruitment, this approach would still not have been particularly useful. Online or remote methods of recruitment could have been used in lieu of face-to-face recruitment, accepting some loss in data completeness as not all physical data could be collected in this way.

#### Retention and data completeness

Participant retention and data completeness in the pilot BABY1000 study was most challenging postpartum, when contact with researchers was no longer aligned with routine healthcare visits. The COVID-19 pandemic also affected postpartum visits most, since most participants were recruited before 2020. Whilst 80% of participants were retained in the study throughout pregnancy, 35% were lost to follow up or withdrew in the two years following their child’s birth, with the majority not having “complete” data across the entire study period.

Improving ease of participation would likely have improved BABY1000. Although participants could provide some samples and complete questionnaires without face-to-face contact, ensuring visits were more convenient with greater scheduling flexibility and accessibility (e.g. parking permits, home visits) [37] and enhancing ease of remote participation (e.g. via a centralised data collection and personalised reminder system, such as an app) [38], may have increased retention and data completeness, reduced the burden on researchers, and allowed the study team to undertake process evaluations with feasibility data in real-time.

Further, personalised contact between the participant and study team between visits (especially those scheduled with months to years apart) or when face-to-face visits are not possible would likely have maintained engagement and connection to the study and its team [39]. Practical examples of this could include individualised cards or messages (e.g. around the child’s first birthday), “check-in” messages, and messages of gratitude or achievement when a study checkpoint had been accomplished. Participants in our study and other birth cohorts [40–43] also reported that a motivator to continue with study was the belief that their involvement would help others, but felt they wanted more information about their contribution and study outcomes. Taken together, this suggests that utilising strategies such as study updates or newsletters, alongside research updates, could have improved retention. BABY1000 used Facebook intermittently, however due to financial and human resourcing limitations, this was underutilised. This was similarly reflected by researchers from the Raine Study in Western Australia, where participants felt Facebook would be helpful to maintain engagement [44]. Appropriately using social media alongside facilitating opportunities to build inter-participant and researcher-participant relationships (e.g. face-to-face events, and collaboration on sub-study design via consumer advisory boards) were described as ways of fostering identity, connection and pride with being part of the study [44].

#### Study cost–efficiency

Funding concerns, as with all research projects, ultimately affect every aspect of feasibility. As Manolio and Collins described in their commentary, large prospective cohorts require “nearly unprecedented levels of cost-efficiency” (pg. 2291) to assess the variety of complex factors influencing health and disease [45]. Limited funding and unforeseen adversity due to the COVID-19 pandemic impacted the capacity of BABY1000 to invest in building relationships with participants, and, importantly, adapt to new requirements during the pandemic. Large periods of time were spent on manual tasks (e.g. reminding participants to complete tasks and manual data entry) that could have been improved with earlier investment in technological systems (e.g. an app to send out questionnaires and reminders) or otherwise outsourced (e.g. DNA extraction from stool). In settings with comprehensive medical records, integrating routinely collected and recorded health data with study-specific data should be explored to enhance data completeness and cost-effectiveness with minimal participant input [45]. Ultimately, to maintain participant retention and maximise data completeness, resources should be tailored to what is identified a priori as most important and to where attrition can be anticipated, e.g., in the postpartum period. Future cohorts should carefully consider their research objectives, considering both available funding and human resources in order to ensure research progress within clear constraints.

### Acceptability of BABY1000 and recommendations for future research

Our qualitative and quantitative data regarding the acceptability showed that study processes, including collection of samples, measures, and questionnaire data, and interactions with researchers, were largely acceptable and/or comfortable. Given the circumstances in which this sub-study was conducted, feedback received regarding the BABY1000 pilot study also often related to the impact of COVID-19 on study participation and on participants’ personal lives. Upon reflection, soliciting acceptability feedback early, during and after study completion would be ideal to improve study design both in process, and for research thereafter.

The least acceptable sample collected in BABY1000 was stool, with approximately one third of participants reporting feeling neutral or uncomfortable, similar to another pilot birth cohort in the United Kingdom involving 171 women [46]. In a 2021 study from The Netherlands (*n* = 780), clearly explained instructions, a rationale behind sample collection, and open communication regarding study results improved willingness to provide stool samples for research [47]. Despite increasing popularity of examining the gut microbiota, response rates or acceptability of stool sampling in pregnant women is rarely reported. Future research should continue to interrogate the acceptability of and barriers to stool sampling, particularly given the contribution of this research to our understanding of health and disease [48].

Feedback on study questionnaires was also mixed. Whilst participants understood their value and often found questionnaires interesting, many felt questionnaires were laborious and/or inaccurately reflected nuances in their health behaviours. For example, dietary intake is difficult to capture accurately via food frequency questionnaire (FFQ) but is offset by a relative ease of administration compared to other dietary assessment methods [49]. FFQs remain valid and reproducible tools if applied in similar populations, as used in BABY1000 with the AES [50]. Since questionnaires can provide valuable research data and are relatively inexpensive and simple to administer, future studies should consider shorter, more frequent questionnaire administration via online platforms with embedded processes for reminders, and/or the offer to facilitate questionnaire completion via telephone with a researcher.

More frequent interactions with participants in providing instruction, feedback or support were also suggested by participants, particularly in the postpartum period (where study visits were months to years apart, compared to more frequent visits during pregnancy). As previously reported in a study involving pregnant women [51], multiple interactions between research teams and participants helped to build rapport, trust and commitment to the study, enhancing acceptability and likely feasibility by improving retention and data completeness.

### Limitations

This sub-study provides valuable feasibility and acceptability learnings for future birth cohort study design but was not without limitations. BABY1000 participants spoke English and had low-risk pregnancies, were more highly educated, and slightly older than national birthing population data [52]. Due to these lower sociodemographic “risks” and a relatively small sample size, generalisability of results to other groups, such as those from diverse social, economic and language backgrounds (including Aboriginal and Torres Strait Islander peoples), those living in rural or remote Australia, and/or women receiving alternative models of maternity care (e.g. private obstetric or midwifery care), may be limited. Notably, parents who participated in the acceptability questionnaire and FGDs did so in addition to routine data collection, which may reflect a sample of particularly motivated and/or altruistic subjects. Further, whilst our STAI-6 results were similar to those previously published concerning women in the perinatal period [53, 54], they represent a snapshot of relatively common feelings of anxiety at the time of administration and may have differed for groups of women exposed to different pandemic containment measures, and/or personal circumstances.

## Conclusion

The BABY1000 pilot study demonstrates that it is feasible and acceptable to collect a range of biological, questionnaire, and health indicator data from early pregnancy to two years of age, but that achieving data completeness is challenging. The COVID-19 pandemic and associated public health orders significantly impacted both measures of study feasibility and acceptability. This should be considered when evaluating results from BABY1000 and similar research conducted across the pandemic, and in designing future cohorts. Recommendations regarding recruitment, retention, data completeness and study cost-effectiveness are proposed to improve the design and resilience of future birth cohort studies.

## Supplementary Information

Supplementary Material 1.

## Data Availability

No datasets were generated or analysed during the current study.
