# Supplementary material for: Feasibility and acceptability of conducting a birth cohort study during the COVID-19 pandemic: a mixed-methods study
Source: BMC Pregnancy Childbirth. 2025 Jul 16;25:767. doi: 10.1186/s12884-025-07850-3 (PMC12265286; doi:10.1186/s12884-025-07850-3)
Supplement: Supplementary file 1 — Supplementary Material 1. [file 12884_2025_7850_MOESM1_ESM.docx]

**Supplementary Material 1 – Online Questionnaire**

**The BABY1000 pilot study participant experience and impact of the COVID-19 pandemic – Questionnaire open to all participants**

**BABY1000 participation questions**

1. How easy or difficult was it for you to participate in BABY1000?

- Likert scale from ‘Easy’ to ‘Very difficult’ (0 – 5)

1. How comfortable were you with providing the samples requested of you as a participant?

Please select N/A if you did not provide the sample listed.

*Blood samples*

- Likert scale from ‘Very comfortable’ to ‘Very uncomfortable’ (0 – 5)
- N/A

*Stool samples (mother)*

- Likert scale from ‘Very comfortable’ to ‘Very uncomfortable’ (0 – 5)
- N/A

*Stool samples (infant)*

- Likert scale from ‘Very comfortable’ to ‘Very uncomfortable’ (0 – 5)
- N/A

*Saliva samples*

- Likert scale from ‘Very comfortable’ to ‘Very uncomfortable’ (0 – 5)
- N/A

1. How comfortable were you with providing the measures requested of you as a participant?

Please select N/A if you did not provide the measure listed.

*Height and weight (mother)*

- Likert scale from ‘Very comfortable’ to ‘Very uncomfortable’ (0 – 5)
- N/A

*Weight and length (infant)*

- Likert scale from ‘Very comfortable’ to ‘Very uncomfortable’ (0 – 5)
- N/A

*Maternal body composition using the BodPod machine*

- Likert scale from ‘Very comfortable’ to ‘Very uncomfortable’ (0 – 5)
- N/A

*Infant body composition using the PeaPod machine*

- Likert scale from ‘Very comfortable’ to ‘Very uncomfortable’ (0 – 5)
- N/A

*Toddler body composition using the BodPod machine (at 24 months)*

- Likert scale from ‘Very comfortable’ to ‘Very uncomfortable’ (0 – 5)
- N/A

*Developmental assessment (24 months)*

- Likert scale from ‘Very comfortable’ to ‘Very uncomfortable’ (0 – 5)
- N/A

1. How comfortable were you providing information via questionnaire related to personal information (demographics, medication use, mental health, physical activity, etc)?

Please select N/A if you did not provide this information.

- Likert scale from ‘Very comfortable’ to ‘Very uncomfortable’ (0 – 5)
- N/A

Open field for additional comments

1. How comfortable were you providing dietary information online (Australian Eating Survey)?

Please select N/A if you did not provide this information.

- Likert scale from ‘Very comfortable’ to ‘Very uncomfortable’ (0 – 5)
- N/A

Open field for additional comments

1. Most questionnaires in the BABY1000 pilot study were administered on paper. We are interested to know your preferred way of completing questionnaires. Please indicate your preferred method of completing questionnaires:

- Electronically at home
- Electronically at the time of your visit
- On paper at home
- On paper at the time of your visit

1. Can you tell us about how appropriate you felt about the length of the questionnaires?

- Too long
- Too short
- Just right

Please feel free to comment anything further related to the questionnaires administered as part of the BABY1000 study. [*Open field for additional comments]*

1. How comfortable were you with receiving information and/or reminders from BABY1000 researcher staff regarding sample collection or questionnaire completion via the following?

*Phone*

- Likert scale from ‘Very comfortable’ to ‘Very uncomfortable’ (0 – 5)
- N/A

*Email*

- Likert scale from ‘Very comfortable’ to ‘Very uncomfortable’ (0 – 5)
- N/A

*Text*

- Likert scale from ‘Very comfortable’ to ‘Very uncomfortable’ (0 – 5)
- N/A

Open field for additional comments

1. How much did the COVID-19 pandemic affect your willingness or ability to participate in the BABY1000 study?

- Likert scale from ‘Very little’ to ‘Very much’ (0 – 5)

Do you have any further comments relating to how your participation in the BABY1000 study was affected by the COVID-19 pandemic? _____________

***Individual COVID-19 impact questions***

*(Not included in this manuscript, therefore removed from this supplement)*

**Spielberger State-Trait Inventory (6-item Short Form)**

A number of statements which people use to describe themselves are given below. Please read each statement and indicate how you feel right now. There are no right or wrong answers. Do not spend too much time on any statement but give the answer which describes your current feeling the best. *(4-point scale from ‘Not at all’ to ‘Very much’)*

1. I feel calm
2. I am tense
3. I am upset
4. I am relaxed
5. I feel content
6. I am worried

*Thank you very much for completing this questionnaire.*

*Please specify if you would be willing to contribute to an online focus group to explore your responses further. We will be contacting interested participants to offer an opportunity to participate in further discussion, followed by a question-and-answer session on infant and toddler feeding with a paediatric dietitian.*

- *Yes, I am interested in participating in an online focus group discussion*
- *No, I am not interested*

**Supplementary Table 1.** Maternal and infant biological samples, measurements, and questionnaires across the BABY1000 pilot study timeline. Darker shaded cells indicate when the respective data was collected.

|  | **PRE*** | **PREGNANCY** | | | | **CHILD** | | | | **MOTHER (POSTPARTUM)** | |
| --- | --- | --- | --- | --- | --- | --- | --- | --- | --- | --- | --- |
|  |  | **12w** | **20w** | **28w** | **36w** | **6w** | **6mo** | **12mo** | **24mo** | **6w** | **24mo** |
| ***Biological samples*** |  | | | | | | | | | | |
| Saliva cortisol |  |  |  |  |  |  |  |  |  |  |  |
| Saliva microbiome |  |  |  |  |  |  |  |  |  |  |  |
| Stool microbiome |  |  |  |  |  |  | ^ |  |  |  |  |
| Buccal swab |  |  |  |  |  |  |  |  |  |  |  |
| Blood |  |  |  |  |  |  |  |  |  |  |  |
| ***Clinical measurements*** |  | | | | | | | | | | |
| Blood pressure |  |  |  |  |  |  |  |  |  |  |  |
| Body composition (BodPod) |  |  |  |  |  |  |  |  |  |  |  |
| Body composition (PeaPod) |  |  |  |  |  |  |  |  |  |  |  |
| Anthropometry |  |  |  |  |  |  | ^ | ^ |  |  |  |
| Aortic Intima Media Thickness |  |  |  |  |  |  |  |  |  |  |  |
| ***Questionnaires*** |  | | | | | | | | | | |
| Demographics |  |  |  |  |  |  |  |  |  |  |  |
| Diet – Food Frequency (AES) |  |  |  |  |  |  |  |  |  |  | + |
| Diet – Infant Feeding Survey |  |  |  |  |  |  |  |  |  |  |  |
| Diet – 24-hour recall |  |  |  |  |  |  | ^ |  |  |  |  |
| Anxiety (STAI-6) |  |  |  |  |  |  |  |  |  |  |  |
| Depression (Edinburgh Scale) |  |  |  |  |  |  |  |  |  |  |  |
| Depression (DASS-21) |  |  |  |  |  |  |  |  |  |  |  |
| Physical activity (IPAQ) |  |  |  |  |  |  |  |  |  |  |  |
| Perceived social support |  |  |  |  |  |  |  |  |  |  |  |
| Functional health literacy |  |  |  |  |  |  |  |  |  |  |  |
| Life stressor checklist |  |  |  |  |  |  |  |  |  |  |  |
| Attitudes to breastfeeding |  |  |  |  |  |  |  |  |  |  |  |
| Sleeping practices |  |  |  |  |  |  |  |  |  |  |  |
| Ages and Stages Questionnaire |  |  |  |  |  |  |  |  |  |  |  |
| Development (PARCA-R) |  |  |  |  |  |  |  |  |  |  |  |
| Feasibility and acceptability ~ |  |  |  |  |  |  |  |  |  |  |  |
| Impact of COVID-19 ~ |  |  |  |  |  |  |  |  |  |  |  |

**Legend:** PRE* = preconception, measured if possible. ^ = subset of children only. + = partners also included in this measurement. AES = Australian Eating Survey. STAI-6 = 6-item State–Trait Anxiety Inventory. DASS-21 = Depression, Anxiety and Stress Scale. PARCA-R = Parent Report of Children’s Abilities-Revised questionnaire. ~ = focus group also conducted, following from questionnaire. Participants were invited if their child was between 6 and 36 months of age (not completed at a specific point in the study timeline).

**Supplementary Table 2.** Illustrative quotes from focus group discussions in relation to acceptability of the BABY1000 pilot study.

| Theme | Codes | Quotes | Key participant characteristics (age range, year child in study was born, total number of children) |
| --- | --- | --- | --- |
| Sample collection | Samples were easy to collect and/or return | “…because the kits were like always the same, like it's always saliva or stool, so I was very confident in how to do it because I was shown by someone at the hospital.” | <30 years, 2020, 1 |
|  |  | “Sending the collection kits in the mail was helpful during COVID.” | 30–34 years, 2020, 1 |
|  | Stool sampling was unpleasant | “When your stomach's already little bit sensitive [during pregnancy], it’s not your favourite activity!” | 30–34 years, 2019, 1 |
|  |  | “The only issue I had was trying to collect a stool sample and I refused. But other than that, it was all good...” | 30–34 years, 2019, 1 |
|  |  | “…it was a bit embarrassing to bring my own stool sample in.” | <30 years, 2021, 1 |
|  | Further instructions or feedback was needed | “Maybe a little short summary with the kits would have been good … like a timeline of when I'm supposed to collect the poo, or the saliva… it probably was there but you had to go through the information, and I mean, with a small baby, it can be difficult... it's fine for us to read all the paperwork but it isn't when we have a baby screaming at that time!” | 30–34 years, 2020, 1 |
|  |  | “Often with the samples that we sent off, questionnaires or scans, there wasn't a lot of feedback, personally, and so I would have liked, maybe a little bit more information about all the things that I was sending off and how my health was, and the baby's health was.” | 35–39 years, 2018, 1 |
| Questionnaire completion | Questionnaires were too long or difficult to complete | “Lockdown restrictions early on meant I couldn't get any extra help around the house … to have time to do other things (like filling out questionnaires from pilot studies!)” | 30–34 years, 2020, 1 |
|  |  | “For me, the I remember thing I hated the most was there was an online diet questionnaire that we did, and it was split in two, so we kept getting sent one for the baby in one for us. And it confused the hell out of me … I’d fill it out, thinking it was me, but I think they were referring to the child, and I just think it would have been better to have one questionnaire about diet and then spell out, sort of, “this section refers to the baby, this section refers to you” – that would have made things a lot easier for me.” | 35–39 years, 2018, 1 |
|  | Questionnaires were not always administered at the intended time | “I had to sort of backtrack, which was impossible really, because he fluctuated so much with his eating as well... it was very, very difficult to fill out those surveys when I was backtracking and trying to remember where he was at that particular stage.” | 30–34 years, 2018, 2 |
|  | Preference for online questionnaire delivery | “Maybe a mobile friendly way to fill out those surveys is an idea. I remember trying to fill it out on my phone and I couldn't read some of the questions and so, then I opted to do it later, and then you know later became later and later… there's a lot of me sitting in doctor's waiting rooms and things like that, where it would have been good to fill out the survey on my phone, so maybe an app would make that easier.” | 30–34 years, 2019, 1 |
|  |  | “…too long for paper questionnaires. If they were electronic would have been fine probably! | 30–34 years, 2020, 1 |
|  | Questionnaires were interesting to complete | “It made me learn about my baby a little bit more, because some things I do not do on an everyday basis so it was that time where I could like you know focus on my baby, count her teeth, think about what I'm feeding her, you know, like more consciously.” | <30 years, 2020, 1 |
| Communication from researchers | Information provided from researchers was clear and respectful | “I felt like everything was very well communicated to me. I knew exactly what was happening. I'm very much like I put everything in my diary, and I had a few extra appointments and things I had to go to so there was a lot of going to the hospital and I found that ... it coincided well with my other appointments.” | 35–39 years, 2, 2020 |
|  |  | “Communication from the staff was great. Just reminding and then confirming the appointments. The communication from the staff was really great, especially like once you're in the hospital where you have to find where you will be, they made the process kind of like smoother, a lot smoother.” | 35–39 years, 2019, 2 |
|  | Information, feedback, or instructions could have been improved or repeated | “I felt like there was a lot of measurements taken but I wasn't really sure what they meant or whether that was a healthy score...” | 30–34 years, 2019, 1 |
|  |  | “I definitely needed reminders, because yeah, my memory was just a total sieve at that time!” | 30–34 years, 2020, 1 |
|  |  | “I think it might be interesting to receive updates on what's happening with the research, like at what stage, it is like is it progressing is it on pause or anything… we only hear from the team when they need something from us, right, but it would be nice to hear from the team with some updates.” | <30 years, 2020, 1 |
|  | Communication around protocol changes due to COVID-19 restrictions could have been improved | “I only had interactions with the team of BABY1000 during my pregnancy… after that there was no contact with me, I mean other than when they sent the surveys to complete, but nobody has seen my baby or anything, since I mean she was born … but still like there was no appointments or anything to follow up, because of COVID.” | 30–34 years, 2019, 2 |
|  |  | “I don't know how the study changed to accommodate COVID-19 restrictions… I don't think I followed the standard course, because of COVID… I do think I received an email about COVID-19 and how it meant part of the data collection had been cancelled or changed but it's a faint memory.” | 30–34 years, 2019, 2 |
| Benefits of participation | Access to health professionals and individualised feedback | “I really enjoyed the consultation with the paediatrician because there was a bit of feedback… having an extra person to give you feedback on how you're progressing with your baby, I like that a lot.” | 30–34 years, 2020, 1 |
|  |  | “I liked it especially for my first child and I like the extra contact with someone in the hospital, because the whole time everything is new and you're not sure if everything is going well, and it was just nice to have someone extra to talk to during the process.” | 30–34 years, 2020, 1 |
|  |  | “I think going through and doing the BABY1000 was great, so it's almost like you're seeing a private obstetrician and so, if you have any questions or if anything’s going wrong, with blood tests and everything, they’re going to let you know, so I thought that was fantastic.” | 35–39 years, 2019, 1 |
|  | Access to additional scans in pregnancy | “I like the extra scans, you know, the reassurance of, you know, the opportunity to hear his heartbeat.” | 35–39 years, 2019, 2 |
|  |  | “I really liked having the scans… because it was so much more efficient compared to the I guess the main hospital, like the wait times were just so much shorter, that was a massive advantage for me.” | 30–34 years, 2018, 1 |
|  | Contributing to research | “I liked idea of contributing to something...that my contribution might change something in the future.” | <30 years, 2020, 1 |
|  |  | “I really liked it. I didn't think it was any more or much more work than what we were doing anyway, as in going into scans and appointments and stuff. I guess I just liked that it was doing some kind of research, and helping somebody right?” | 30–34 years, 2019, 1 |
| Partner involvement | Partner involvement would be desired, acceptable, and feasible | “For us, I was I like research, so I was actually the one that suggested [participant’s name] do it. I was pretty keen on being involved, I think, anyway. So yeah, like I think for me like there would be value in collecting data from the partner as well.” | <30 years, 2021, 1 – partner of participant |
|  |  | “I think my partner would happily participate, but he's I guess research minded so would see the great benefits [of participating].” | 30–34 years, 2018, 1 |
|  | Partner involvement would *not* be desired, acceptable, and/or feasible | “I’d say forget it. Forget it! I think I do all the admin for everything, and even just that last dietary question, because I cook and I serve, and I see what everyone eats, I just did it. Thank God he wasn’t involved the whole way through. I would have just done things on his behalf... I had to do his questionnaire online for him. I couldn't do that for two years, worth of data collection.” | 30–34 years, 2019, 2 |
|  |  | “I think he would be happy with the questions, but not with like sample collections or anything like that... I will have to keep reminding him and keep everything organised to remind him... because he wouldn't have the motivation or interest.” | <30 years, 2020, 1 |
| COVID impact | Protocol changes due to COVID-19 were undesirable | “I feel like COVID robbed us of a whole lot of the second half of the of what was meant to happen, you know, since they were born, I have filled out surveys and I’ve seen one person… everything was locked down, but it's like we missed a whole lot of that assessment stuff that I really would have liked... it's not the kind of stuff that you can pick up later because it's age dependent, and those ages have gone now.” | 30–34 years, 2019, 3 |
|  |  | “I didn't get the extra scan; I think that was a COVID adjustment? So, I was a bit like “Ohh”, that was a shame, because I had really liked that with my first involvement. Yeah, so that could just a point that that was a disappointment, I suppose, second time around.” | 30–34 years, 2019, 2 |
|  |  | “COVID didn't impact my willingness to participate, but I feel we missed a lot of the post birth assessment. My babies were born on the first day of lockdown in Australia… We have seen only one person since they were born 18 months ago (from my memory, the early days are hazy). I feel we missed out on a few things that I would have been very interested to participate in, and to have results for my children from. There are also things on the lists that should be upcoming, and I hope we are able to participate fully…” | ≥40 years, 2020, 3 |
|  | Reluctance to attend a healthcare setting for study visits | “It was pretty easy with the surveys and everything in the beginning, and then, once COVID hit that kind of died down and then all of a sudden, I was still freaking out about COVID, and you guys wanted me to come in and I couldn't. Just because I was scared of just bringing my little boy into the hospital environment...” | ≥40 years, 2020, 1 |
|  |  | “Despite wanting to participate in the 2-year development assessment in person, I was not comfortable to attend the hospital due to COVID risk.” | 30–34 years, 2019, 2 |
|  | Additional precautions (PCR testing) were uncomfortable / burdensome | “Extra COVID tests for myself & child. Visit took a bit longer as needed to go check in at hospital then go to [study centre] and carting child around makes everything take even longer. Hospital & centre felt a bit eery with no-one around and all the extra check points.” | 30–34 years, 2019, 1 |
